# Recovery in Special Emphasis Populations

**DOI:** 10.35946/arcr.v40.3.05

**Published:** 2020-12-10

**Authors:** Eric F. Wagner, Julie A. Baldwin

**Affiliations:** 1 Robert Stempel College of Public Health & Social Work, Community-Based Research Institute, and National Institute on Minority Health and Health Disparities (NIMHD) Research Center in a Minority Institution (RCMI), Florida International University, Miami, Florida; 2 Center for Health Equity Research and NIMHD RCMI Southwest Health Equity Research Collaborative, Northern Arizona University, Flagstaff, Arizona

**Keywords:** alcohol-related disorders, alcoholism, minority health, health status disparities, Alcoholics Anonymous, social justice, alcohol, sexual and gender minorities

## Abstract

Special emphasis populations in the current context can be defined as groups experiencing health disparities resulting in elevated risk to health, safety, and well-being from drinking alcohol. Individuals from marginalized minority populations often encounter barriers to accessing and receiving effective alcohol treatment due to social inequities and disadvantaged life contexts, which also may adversely affect recovery from alcohol use disorder (AUD). Recovery from AUD often involves the adoption of a stable non-drinking lifestyle (sobriety), increased health and well-being, and increased social connection. Although there has been considerable work on AUD epidemiology among special emphasis populations, little research exists directly examining recovery among racial/ethnic minority populations and/or sexual and gender minority populations. The current narrative review hopes to spark scholarly interest in this critically neglected area. This article opens with a review of special emphasis populations and their alcohol-related risks. Next, definitions of recovery, Alcoholics Anonymous, and culturally adapted recovery models for racial/ethnic minority populations are explored. This is followed by a discussion of factors that may particularly influence recovery among marginalized minority populations. This narrative review concludes with a discussion of research priorities for promoting health equity through studies focused on understanding and supporting recovery from AUD among marginalized minority populations.

## INTRODUCTION

The National Institute on Alcohol Abuse and Alcoholism (NIAAA) defines special emphasis populations as “groups who face particular risks from drinking alcohol based on personal characteristics such as age or gender.”^1^ Underage youth, emerging adults (ages 18 to 28), older adults (age 65 and older), women, individuals experiencing co-occurring disorders, and ethnic and racial minorities are special emphasis populations highlighted by NIAAA. Additional special emphasis populations at heightened risk for AUD include sexual minorities,^2^–^4^ individuals with justice system involvement,^5^–^10^ homeless persons,^11^ and former foster care emerging adults.^12^

### Underage Youth

Underage youth are a special emphasis population given the ubiquity and inherent danger of underage drinking, as well as the status illegality of drinking among minors. By 12th grade, most Americans will have consumed alcohol, half will have consumed alcohol in the past year, and 1 out of 7 will have had five or more drinks in a row in the past 2 weeks.^13^ Underage drinking is remarkably dangerous, carrying with it substantial risk to the health, safety, and well-being of teenagers and those around them.

### Emerging Adults

Emerging adults are distinguished by the highest risk for alcohol and drug use problems of any age group.^14^ More than a third of emerging adults report binge drinking during the past 2 weeks; those attending college are at higher risk for drinking problems than those not attending college, and collegians who participate in Greek letter organizations (“Greek life”) are at especially high risk.^15^

### Older Adults

NIAAA considers older adults (age 65 and older) a special emphasis population because many drink despite (1) age-related increases in sensitivity to alcohol, (2) health problems complicated by drinking, and (3) using medications that interact poorly with alcohol.^16^ Moreover, drinking problems among older adults often are associated with factors unique to senior adulthood, such as aging-related health worries, boredom after retirement, the death of friends and loved ones, shame about drinking, and the justification that drinking is harmless to others.

### Individuals With Co-Occurring Disorders

Co-occurring disorders alongside AUD are common, and individuals with co-occurring disorders are a special emphasis population given the complexities associated with treating AUD alongside other disorders. People with drinking problems are at heightened risk for psychiatric problems (i.e., anxiety disorders, depressive disorders, bipolar disorders, attention-deficit/hyperactivity disorder, borderline personality disorder, antisocial personality disorder, schizophrenia); problems with the use of other drugs in addition to alcohol; and physical problems and conditions (e.g., liver disease, HIV/AIDS, alcohol-related cancers). This comorbidity is a product of genetic vulnerabilities, epigenetics, neurobiology, environment, exposure to stress, and trauma. As highlighted by NIAAA, having co-occurring disorders is associated with greater alcohol problem severity;^17^ moreover, it complicates the treatment of AUD, which for optimal effectiveness must be integrated with treatment(s) for co-occurring disorders.

### Women

NIAAA regards women as a special emphasis population given the higher risk of certain alcohol-related negative consequences compared to men, such as liver damage, heart disease, brain damage, and breast cancer.^18^ Moreover, women are a special emphasis group due to the issues of drinking during pregnancy and fetal alcohol exposure. In general, women report more problems related to physical and mental health as well as more past trauma and abuse (physical and sexual). Notably, women are more likely than men to begin using alcohol and drugs after a specific traumatic event and to suffer from post-traumatic stress disorder.^19^ Key principles in women’s recovery include addressing any experiences of trauma, including incest and rape, fears of losing their children, and parenting challenges and efficacy.^20^–^23^

### Racial and Ethnic Minorities

NIAAA^24^ points out “certain ethnic and racial minorities as well as other underserved populations experience more negative consequences of illness and premature death than other groups,” noting disparities affecting (1) Hispanics/Latinx, (2) Blacks, and (3) Native Americans. The life contexts of racial and ethnic minority individuals with AUD are likely to include more economic hardship, stress, systemic discrimination and prejudice, and compounded disadvantage, as well as fewer recovery resources and supports, compared to the life contexts of non-Hispanic White individuals with AUD. The marginalization associated with racial/ethnic minority status produces enduring and significant challenges to recovery for such individuals.

The remainder of this narrative review focuses on individuals from marginalized minority groups in the recovery phase of their drinking careers, with particular attention to what may distinguish recovery challenges experienced by minority populations from those experienced by majority populations. It should be noted that rigorous empirical studies directly investigating recovery among any marginalized minority population(s) are absent from the literature; in contrast, considerable research has been conducted on the epidemiology of AUD and alcohol-related negative consequences among minority populations. The current narrative review draws heavily on that epidemiological work and extends it to recovery by: (1) examining what is known about recovery among minority populations; (2) identifying factors and mechanisms that especially may impact recovery among minority populations; and (3) suggesting avenues for additional research.

## DEFINING RECOVERY AMONG SPECIAL EMPHASIS POPULATIONS

Despite widespread common usage of the term “recovery,” obtaining expert consensus on the essential elements for defining recovery from AUD has proved challenging. The Substance Abuse and Mental Health Services Administration (SAMHSA) defines recovery as “a process of change through which individuals improve their health and wellness, live a self-directed life, and strive to reach their full potential.”^25^ Moreover, SAMHSA conceptualizes recovery along four dimensions: health, home, purpose, and community relationships/social networks. The Betty Ford Institute Consensus Panel defines recovery as “a voluntarily maintained lifestyle” characterized by sobriety (abstinence from alcohol and nonprescribed drugs), personal health (improved quality of personal life), and citizenship (respect for others).^26^ William White defines recovery as “the experience (a process and a sustained status) through which individuals, families, and communities impacted by severe alcohol and other drug (AOD) problems utilize internal and external resources to voluntarily resolve these problems, heal the wounds inflicted by AOD-related problems, actively manage their continued vulnerability to such problems, and develop a healthy, productive, and meaningful life.”^27^ Despite considerable overlap among these three influential recovery definitions, they differ in meaningful ways with one another (e.g., whether recovery is voluntary; whether recovery means enduring vulnerability).

Kaskutas et al. reached out to adults in recovery (*n* = 9,341) and asked them how they defined recovery.^28^ Responses revealed three factors: (1) “abstinence” (no use of alcohol); (2) “essential recovery” (being honest with oneself, handling negative feelings without drinking or using, enjoying life without drinking or using); and (3) “enriched recovery” (ongoing growth and development, reacting to life in a more balanced way, taking responsibility). In post hoc analyses, Kaskutas et al. examined possible variation by race/ethnicity and education in definitions of recovery, and found almost none. Notably, adults in recovery with less than a college degree or from racial/ethnic minorities were less likely than their counterparts to emphasize abstinence in defining recovery, and more likely to emphasize the essential recovery and enriched recovery factors. Overall, these differences were slight, suggesting considerable overlap in definitions of recovery among and across minority and majority populations in recovery.

## PARTICIPATION IN ALCOHOLICS ANONYMOUS BY MINORITY POPULATIONS

Participation in formal alcohol treatment typically precedes entering recovery. Kaskutas et al. found that 96% of adults self-identifying as being in recovery had received treatment for AUD.^28^ The overwhelming majority of alcohol treatment programs in the United States incorporate 12-step elements and promote participation in Alcoholics Anonymous (AA) as an aid to recovery. AA was founded by non-Hispanic White men in the 1930s, and historically most AA members in the United States have been non-Hispanic White; over time, AA members have become much more diverse, reflecting the increasing demographic diversity of the U.S. population.

Concerned that AA’s non-Hispanic White origins might be a barrier to AA participation for minority populations, Tonnigan, Connors, and Miller reviewed the literature and concluded: (1) AA is well known and well liked among minority populations; (2) minority populations are less likely to avail themselves of AA compared to nonminority populations; and, (3) minority populations are as likely to benefit from AA as nonminority populations.^29^ In the 2 decades since the published review by Tonnigan et al., AA has grown substantially in the number of interest groups, meetings, conventions, and program resources designed especially for minority populations in recovery from AUD (e.g., http://gal-aa.org/ for gays and lesbians; https://naigso-aa.org/ for Native Americans).

### AA Special Emphasis Group Adaptation: The Native American Wellbriety Movement

Some minority populations have adapted AA literature, rituals, and materials to increase AA’s appeal, as well as cultural and linguistic appropriateness, for members of their communities. Beginning in the 1960s, AA has been steadily adapted by American Indian communities, culminating in the Wellbriety movement.^30^ Wellbriety frames AUD from an American Indian perspective, where all things are holistically connected, and there is no separation between the individual, family, and tribe. Moreover, the fourth edition of the Big Book of Alcoholics Anonymous^31^ has revised and updated its depictions of Native American culture, and a growing number of Native American meetings are registering with the AA General Services Office ( https://naigso-aa.org/).

Despite the advances of the Wellbriety Movement, the relative dearth of AUD treatment and aftercare approaches congruent with Native American cultural values, beliefs, and traditions remains a major barrier to recovery from AUD for Native Americans.^32^,^33^ Tradition-based Native American practices that may be incorporated into AUD treatment and recovery include: Sweat ceremonies, a cultural practice usually performed in a lodge that uses heat and steam to cleanse toxins from the mind, body, and spirit; smudging or the burning of sacred herbs to purify people and places; the use of ceremonial drums and songs; Talking Circles; traditional healers; and Elder teachings.^34^ Additionally, historical trauma impinges upon Native Americans’ successful recovery from AUD. Brave Heart notes: “Historical trauma, also referred to as a cumulative trauma, soul wound, and intergeneration trauma, refers to the cumulative emotional and psychological harm experienced throughout an individual’s life span and through subsequent generations.”^35^ Historical trauma is the cumulative result of centuries of subjugation, racism and discrimination, genocidal violence, segregation, and systemic oppression inflicted upon Native Americans. Incorporating tradition-based practices, and holistic concepts of wellness and community-based recovery support, can help contextualize and ameliorate the impact of historical trauma on recovery from AUD among Native Americans.^32^,^33^

### AA Special Emphasis Group Adaptations: African American and Hispanic

In African American communities, local church-based drug ministries and mutual aid groups often are indigenous sources of services for recovery initiation, stabilization, and maintenance.^36^ Given AA’s Episcopalian roots and its emphasis on congregation and mutual aid, AA integrates relatively easily with church-based recovery support initiatives in African American communities. In immigrant urban Hispanic/Latinx communities in California, *anexos* are an indigenous adaptation of AA, typically catering to male, lower-income, Spanish-speaking immigrants and migrants.^37^,^38^ Residences literally annexed to AA meeting sites, *anexos* originated in Mexico in 1975 as part of the recovery support “24 Hour Movement” (*Movimiento 24 Horas*), and since have spread to Hispanic/Latinx communities in the United States. Although strides have been made toward the cultural and linguistic adaptation of AA by minority groups, these advances have been limited by an emphasis on heterosexual men; thus, a critical next step is the adaptation of AA for minority women and for intersectional individuals with both racial/ethnic and sexual minority status.

## CHALLENGES TO RECOVERY AMONG MINORITY POPULATIONS

Marginalized minority groups possess limited economic and social capital. Such limitations typically result from social and environmental injustices, and often reflect de jure and de facto discrimination.^39^ Both before and during recovery from AUD, the life contexts of minority populations are likely to include more pervasive and enduring hardships, stresses, and disadvantages compared to the life contexts of majority populations.^40^–^47^ Among marginalized minority groups, disadvantaged life contexts are (1) socially determined, (2) a function of social injustices, and (3) the primary causes of health inequities and disparities.^41^,^42^ This means that the long-term elimination of health disparities, including those associated with recovery from AUD, is dependent on social change.

Research has identified a range of socially determined disadvantaged life contexts that significantly impact the course of AUD among minority populations;^40^–^47^ it is very likely that these same social determinants significantly impact recovery from AUD. Key social determinants that may influence recovery among minority populations include:

- Material hardship
- Residential segregation
- Neighborhood crime and disorder
- Alcohol access through nearby alcohol outlets including bars and liquor stores
- Stigma about having problems with alcohol use or having AUD
- Unfair treatment, prejudice, and discrimination
- Disparities in medical care, resulting in more untreated or undertreated medical conditions
- Housing instability
- Unemployment and underemployment
- Personal demoralization
- Lack of culturally and linguistically appropriate recovery support services nearby
- Stress, from multiple and interacting sources

Such inequity in exposure to economically disadvantaged and health-compromising life contexts is a pressing environmental justice issue. Racial/ethnic minority populations are marginalized groups living in lower-income areas; residential segregation by income and race/ethnicity is considered “the most critical distinctive social exposure” driving health disparities.^49^ Research has shown that the associations between environmental risks and AUD are stronger in poorer neighborhoods, suggesting that environmental challenges are a particular threat to recovery among individuals with AUD from low-income communities.^50^ Although successful recovery from AUD can be difficult and tortuous for anyone, successful recovery for someone from a marginalized minority population includes an added layer of socially determined challenges and environmental injustices. Moreover, a sizable number of people in recovery have more than one minority identity (e.g., a Latinx lesbian, a person of color who is incarcerated); individuals with intersectional identities may be especially likely to encounter socially determined challenges to recovery from AUD.

## RECOMMENDATIONS

NIAAA^51^ has identified four research priorities for investigations regarding the dynamics of posttreatment recovery. Two of these priorities speak directly to decreasing health inequities and enhancing knowledge related to recovery from AUD among minority populations. NIAAA notes that studies are needed on (1) “the neurobiological, psychological, environmental, and social factors that influence post-treatment recovery” and (2) “trajectories of recovery in subgroups of people with different cultural and socioeconomic backgrounds, cognitive abilities, and medical histories.” Keeping these two priorities in mind, the following recommendations are offered for future research on recovery from AUD among minority populations:

- Identify modifiable drivers of recovery among vulnerable populations.
- Estimate the contributions of various life context hardships, stresses, and disadvantages to recovery trajectories among minority populations.
- Explore the intersections of various minority identities (e.g., race, ethnicity, socioeconomic status, sex), alongside experiences of discrimination and injustice, vis-à-vis recovery trajectories.
- Examine how (1) minority populations use or adapt AA, (2) AA practices vary among minority populations, and (3) characteristics of minority populations influence the likelihood of benefitting from AA.
- Investigate the critical transition from treatment completion to community-based recovery, and how that affects long-term recovery trajectories among minority populations.
- Compare the utilization and impact of AA versus other recovery support services (e.g., Wellbriety; SMART [Self-Management and Recovery Training], Celebrate Recovery) among minority populations.

## CONCLUSIONS

Rigorous empirical studies of recovery from AUD among minority populations are absent from the literature. Although many individuals from minority populations respond well to alcohol intervention—successfully completing treatment, ending drinking, and starting recovery—minority populations experience numerous challenges and barriers to recovery from AUD. It is very likely social determinants of health disparities significantly impact recovery from AUD among marginalized minority populations (e.g., racial/ethnic minorities, sexual minorities), but this has yet to be directly examined. Thus, there is an urgent need for investigations of recovery among minority populations. Such research is essential for making progress in eliminating alcohol-related health disparities impacting minority populations.
